# Prevalence, Treatment, and Outcomes of Corneal Disease in Aboriginal and Torres Strait Islander Peoples in Australia: A Systematic Review

**DOI:** 10.1111/ceo.70118

**Published:** 2026-04-13

**Authors:** Angus van der Nagel, Daniel Cool, Himal Kandel, Stephanie Watson

**Affiliations:** ^1^ Sydney Medical School, Faculty of Medicine and Health The University of Sydney Sydney Australia

## Abstract

**Background:**

To synthesise available evidence on the prevalence, treatment strategies, and outcomes of corneal disease affecting Aboriginal and Torres Strait Islander peoples in Australia, and to identify gaps in the existing literature.

**Methods:**

A systematic review with narrative synthesis was conducted in accordance with PRISMA 2020 guidelines. PubMed, Embase, Scopus, and the Cochrane Library were searched from January 2010 to February 2025. Eligible studies reported epidemiology and/or treatment outcomes of corneal disease in Indigenous Australians. Due to heterogeneity, meta‐analysis was not performed.

**Results:**

Twenty‐one studies met inclusion criteria. Active trachoma prevalence declined over the past decade from approximately 14%–15% (1–5) to 3%–4% (6–8), with most jurisdictions reporting prevalence below endemic thresholds by 2022, although pockets of higher prevalence persisted in some remote communities (8). Trachomatous trichiasis was higher among Indigenous Australian adults compared with non‐Indigenous Australian adults (~0.4%–0.5% vs. ~0.03%–0.05%) (5, 8, 9). Evidence for non‐trachomatous corneal disease was limited to clinic and hospital‐based cohort studies. These data suggested that pterygium was common among Indigenous Australians, although lacked population‐level prevalence estimates. Hospital‐based keratitis cohorts demonstrated worse visual outcomes among Indigenous patients, with distinct risk factor profiles characterised by lower contact lens use and higher rates of trauma and delayed presentation.

**Conclusions:**

While available data suggest progress in some corneal conditions among Indigenous Australians, major evidence gaps limit reliable estimates for several pathologies. Priorities include strengthening Indigenous‐specific population data and developing prevention and treatment approaches tailored to environmental exposures and access barriers in remote communities.

## 1 Introduction

Corneal disease is a major cause of vision impairment and blindness worldwide, contributing substantially to preventable visual loss when diagnosis and treatment are delayed [1, 2]. Conditions including trachoma, pterygium, and microbial keratitis account for significant ocular morbidity, with corneal opacity responsible for approximately 5% of global blindness [1, 2]. However, the burden and outcomes of corneal disease vary across populations and remain incompletely characterised in high‐risk groups.

Aboriginal and Torres Strait Islander peoples experience disproportionately higher rates of overall vision impairment and blindness than non‐Indigenous Australians, driven by environmental exposure, geographic isolation, and barriers to timely eye care [3]. Corneal disease contributes meaningfully to this inequity, particularly in remote communities, yet uncertainty persists regarding population‐level prevalence, treatment strategies, and outcomes across corneal pathologies in Indigenous Australians.

Existing evidence is fragmented and condition specific. Robust population‐based data are largely limited to trachoma and trichiasis through national surveillance, while evidence for non‐trachomatous corneal disease is predominantly derived from clinic‐ and hospital‐based cohorts. Although pterygium is strongly associated with ultraviolet exposure common in remote Australian settings, Indigenous‐specific prevalence estimates with measures of uncertainty are scarce [4]. Similarly, microbial keratitis and trauma‐related corneal disease are under‐represented in population‐based studies, limiting generalisability and comparative assessment of outcomes. Consequently, existing studies do not adequately address key questions regarding the overall burden of corneal disease or equity of treatment outcomes across conditions.

This systematic review aimed to synthesise available evidence on the prevalence, treatment strategies, and outcomes of corneal disease affecting Aboriginal and Torres Strait Islander peoples in Australia, and to identify gaps in the existing literature.

## 2 Methods

### 2.1 Protocol and Registration

This review followed PRISMA 2020. The protocol was registered with PROSPERO (CRD420250644892).

### 2.2 Eligibility Criteria

We included peer‐reviewed studies reporting the epidemiology and/or treatment of potentially blinding corneal disease in Aboriginal and Torres Strait Islander peoples (either exclusively or as a pre‐specified subgroup with separate analyses). Corneal conditions of interest included: trachoma and trichiasis (WHO simplified grading), corneal opacity/scar, microbial keratitis (bacterial/fungal; clinical ± microbiological confirmation), pterygium, corneal dystrophies, post‐surgical bullous keratopathy, keratoconus, and trauma‐related corneal disease. Broader eye‐health surveys or studies of vision impairment from multiple causes were eligible only where corneal‐specific data were reported separately or could be extracted independently.

We excluded studies without explicit Indigenous data, non‐Australian settings, animal studies, case reports, editorials, and studies where refractive error was the sole cause of vision loss. No language restrictions were applied beyond feasibility (English‐language databases searched).

### 2.3 Information Sources

We searched PubMed, Embase, Scopus, and Cochrane Library from 1 Jan 2010 to 13th February 2025. The 2010 start date was chosen to capture contemporary evidence relevant to current surveillance, treatment pathways, and service‐delivery models, and because it coincided with the introduction of the Australian Trachoma Surveillance Annual Report 2010, the first recurring national surveillance report meeting our eligibility criteria. Landmark pre‐2010 studies were excluded from formal synthesis but were considered narratively in the Discussion for historical context. We also screened reference lists of included studies and relevant surveillance reports to identify additional eligible primary studies.

### 2.4 Search Strategy

Searches combined population terms (‘Indigenous Australians’, ‘Aboriginal Australian*’, ‘Torres Strait Islander*’) with corneal‐disease terms (e.g., ‘corneal disease*’, ‘corneal opacit*’, keratitis, pterygium, trachoma, trichiasis) using Boolean operators and database‐specific subject headings. Searches were limited to human studies and Australian settings. Full strategies are provided in Appendix A—Supplement 1.

### 2.5 Selection Process

Search results were exported to EndNote; duplicates were removed. Two reviewers (AV, DC) independently screened titles/abstracts against eligibility criteria (include/exclude/unclear). Full texts for ‘include’ or ‘unclear’ were retrieved and screened independently. Disagreements were resolved by discussion; a third reviewer adjudicated if needed. Reasons for exclusion at full text were recorded. A PRISMA flow diagram summarises study within Appendix A.

### 2.6 Data Collection Process

Two reviewers independently extracted data using a piloted form (Microsoft Excel), with discrepancies resolved by consensus. When multiple publications arose from the same underlying dataset or surveillance programme, each paper was extracted if it contributed a distinct time point, outcome, or analysis relevant to the review. Overlap between related publications was taken into account during narrative synthesis to avoid inappropriate double counting.

### 2.7 Data Items

For all studies data collected were: citation, jurisdiction, sampling frame (population‐based vs. programme/community surveillance vs. clinic‐audit), study design, study period, population/age, case definitions, outcomes, and authors' key conclusions.

For prevalence/epidemiology data collected were: denominators (examined population), numerators (case counts), screening coverage, and point prevalence with/without 95% CIs (confidence intervals); for serial rounds, we extracted per‐round estimates.

For treatment/outcomes data fields were: intervention type (including programme strategies), comparators, follow‐up, final visual acuity, complications (e.g., corneal perforation, keratoplasty), and patient‐reported outcomes where available.

### 2.8 Study Risk of Bias Assessment

We assessed risk of bias (ROB) using design‐appropriate tools: Hoy et al./JBI (Joanna Briggs Institute) for prevalence, ROBINS‐I (Risk of Bias In Non‐randomised Studies—of Interventions) for non‐randomised intervention/clinical cohorts. Two reviewers judged each domain independently; overall judgements (low/moderate/high or equivalent) were assigned with rationale.

### 2.9 Effect Measures

For prevalence, the effect measure was proportion (%). For outcomes, we abstracted proportions and means with SD (Standard deviation)/CI as reported.

### 2.10 Synthesis Methods

Given heterogeneity in sampling frames, settings, and case definitions, we did not pool across sampling frames. For this reason, meta‐analysis could not be conducted. We used a structured narrative synthesis, grouping by condition, age, jurisdiction, and sampling frame, and summarising direction and consistency of effects. Serial community surveys are presented per round without pooling individuals across years.

### 2.11 Reporting Bias and Certainty Assessment

We explored small study and reporting bias qualitatively (funnel plots were not planned due to sparse, heterogeneous data). We summarised overall certainty of evidence per outcome domain narratively, considering sampling frame, RoB, precision, and consistency.

## 3 Results

The initial database search yielded 2943 studies. After removing duplicates, 2037 studies progressed to title and abstract screening. Most ineligible studies did not discuss Aboriginal or Torres Strait Islander health, and many did not make explicit mention of corneal disease. Forty‐seven studies progressed to full‐text screening, and 21 studies fully met the inclusion criteria and were included in the analysis (see Table 1).

**TABLE 1:** Characteristics of included studies.

Study/report	Design and sampling frame	Location	Population	Corneal condition(s)	Key outcomes
Landers et al. (2010)—blinding trachoma	Clinic‐based cross‐sectional study	NT (very remote)	Indigenous adults ≥ 20 years	Trachoma; trichiasis; corneal opacity	Prevalence and associations of blinding trachoma
Landers et al. (2010)—visual impairment	Clinic‐based cross‐sectional study	NT (very remote)	Indigenous adults ≥ 20 years	Trachoma‐related vision impairment	Causes of bilateral visual impairment and blindness
Lansingh et al. (2010)	Community‐level intervention study	Central Australia	Indigenous children < 15 years	Trachoma	Impact of SAFE‐based strategies
Taylor et al. (2010)—trachoma prevalence	Population‐based cross‐sectional survey	Australia‐wide	Indigenous children 5–15 years; adults ≥ 40 years	Trachoma; trichiasis; corneal opacity	National trachoma prevalence and sequelae
Cowling et al. (2012)—ATSAR 2010	National surveillance report	NT, WA, SA, NSW	Indigenous children 1–14 years; adults ≥ 40 years	Trachoma; trichiasis	TF and TT prevalence, screening coverage, treatment delivery
Lynch et al. (2022)	Serial cross‐sectional screening with PCR	Remote QLD	Indigenous children	Trachoma	TF prevalence; microbiological confirmation
Lynch et al. (2023)	Serial cross‐sectional screening with PCR	Torres Strait Islands	Predominantly Indigenous children	Trachoma	Clinical TF versus PCR discordance
Jaworski et al. (2025)	National surveillance report	NT, WA, SA, NSW	Indigenous children 1–14 years.; adults ≥ 40 years	Trachoma; trichiasis	Updated national prevalence and at‐risk community classification
Dirani et al. (2018)	Population‐based cross‐sectional survey	Australia‐wide	Indigenous adults ≥ 40 years	Trichiasis	TT prevalence
Landers and Henderson (2011)	Clinic‐based cross‐sectional study	NT (very remote)	Indigenous adults ≥ 20 years	Pterygium	Prevalence
McGlacken‐Byrne et al. (2021)	Retrospective clinic audit	Remote WA	84.7% Indigenous	Pterygium	Prevalence by Indigenous status
Richards et al. (2016)	Retrospective hospital cohort	NT	23.4% Indigenous	Microbial keratitis	Risk factors; visual outcomes
Kim et al. (2024)	Retrospective hospital cohort	NT	26% Indigenous	Fungal keratitis	Risk factors; outcomes (not Indigenous‐stratified)
Cowling et al. (2013)—ATSAR 2011	National surveillance report	NT, WA, SA, NSW	Indigenous children 1–14 years; adults ≥ 40 years	Trachoma; trichiasis	TF and TT prevalence, screening coverage, treatment delivery
Cowling et al. (2015)—ATSAR 2012	National surveillance report	NT, WA, SA, NSW	Indigenous children 1–14 years; adults ≥ 40 years	Trachoma; trichiasis	TF and TT prevalence, screening coverage, treatment delivery
Cowling et al. (2016)—ATSAR 2013	National surveillance report	NT, WA, SA, NSW	Indigenous children 1–14 years; adults ≥ 40 years	Trachoma; trichiasis	TF and TT prevalence, screening coverage, treatment delivery
Foreman et al. (2018)	Population‐based cross‐sectional survey	Australia‐wide	Indigenous adults ≥ 40 years	Corneal vision loss	Unilateral impairment and blindness
Taylor et al. (2010)—vision loss	Population‐based cross‐sectional survey	Australia‐wide	Indigenous children 5–15 years; adults ≥ 40 years	Trachoma‐related vision loss	Causes of bilateral low vision and blindness
Landers et al. (2013)	Clinic‐based incidence cohort	NT (very remote)	Indigenous adults ≥ 20 years	Trachoma‐related visual impairment	Incidence of visual impairment due to trachoma
Taylor et al. (2012)	Serial single‐community survey	NT	Indigenous children 5–13 years	Trachoma	Long‐term change in TF prevalence
Liu et al. (2016)	Observational analysis of programme data	NT, WA, SA	Indigenous children 5–9 years	Trachoma	Effect of antibiotic strategies

*Note:* Individual papers are listed separately.

Abbreviations: NSW, New South Wales; NT, Northern Territory; PCR, polymerase chain reaction; SA, South Australia; TF, trachomatous inflammation‐follicular; TT, trachomatous trichiasis; WA, Western Australia.

### 3.1 Study Characteristics

Thirteen of the 21 included papers focused primarily on trachoma or trichiasis, all of which had almost exclusively Indigenous participants, although exact percentages were not always reported. Two papers examined pterygium and two examined infectious keratitis. Four additional papers reported broader causes or rates of visual impairment in Indigenous Australians, but were included only because corneal pathologies were reported separately as extractable subgroups. No eligible studies were identified for other corneal pathologies such as dystrophies or ocular surface neoplasia. No studies directly examined corneal trauma, although trauma was reported as a risk factor for keratitis in two publications.

Included papers comprised five national surveillance reports, four population‐based cross‐sectional surveys, six community‐ or clinic‐based cross‐sectional prevalence studies, two retrospective clinical cohorts, two programme/intervention evaluations, one clinic‐based incidence cohort, and one serial single‐community survey.

Several papers arose from recurring surveillance programmes or shared datasets, but are listed individually in Table 1 for transparency because they contributed distinct time points or outcome analyses.

### 3.2 Risk of Bias Assessment

Risk of bias (RoB) was low to moderate overall for prevalence‐focused studies, particularly those with standardised sampling frames and case definitions (Table 2). National surveillance and survey studies demonstrated the lowest risk in outcome measurement, but variable risk related to representativeness and incomplete reporting of participation rates. Clinic‐based prevalence studies showed a higher risk of selection bias. Retrospective clinical cohorts and intervention studies were judged to have moderate RoB due to confounding and lack of control groups. Full domain‐level RoB assessment is contained within Appendix A—Supplement 2.

**TABLE 2:** Summary of risk of bias of included studies.

Study	Study type	Risk of bias tool	Risk of bias
Landers et al. 2010—blinding trachoma	Clinic‐based cross‐sectional study	Hoy/JBI	Moderate
Landers et al. 2010—visual impairment	Clinic‐based cross‐sectional study	Hoy/JBI	Moderate
Lansingh et al. 2010	Intervention study	ROBINS‐I	Moderate
Taylor et al. 2010—trachoma prevalence	Population survey	Hoy/JBI	Low
Cowling et al. 2012—ATSAR 2010	National surveillance	Hoy/JBI	Moderate
Lynch et al. 2022	Community survey	Hoy/JBI	Moderate
Lynch et al. 2023	Community survey	Hoy/JBI	Moderate
Jaworski et al. 2025	National surveillance	Hoy/JBI	Moderate
Dirani et al. 2018	Population survey	Hoy/JBI	Low
Landers and Henderson 2011	Clinic prevalence	Hoy/JBI	Moderate
McGlacken‐Byrne et al. 2021	Clinic audit	Hoy/JBI	Moderate
Richards et al. 2016	Retrospective cohort	ROBINS‐I	Moderate
Kim et al. 2024	Retrospective cohort	ROBINS‐I	Moderate
Cowling et al. 2013—ATSAR 2011	National surveillance	Hoy/JBI	Moderate
Cowling et al. 2015—ATSAR 2012	National surveillance	Hoy/JBI	Moderate
Cowling et al. 2016—ATSAR 2013	National surveillance	Hoy/JBI	Moderate
Foreman et al. 2018	Population survey	Hoy/JBI	Low
Taylor et al. 2010—vision loss	Population survey	Hoy/JBI	Low
Landers et al. 2013	Clinic‐based incidence cohort	Hoy/JBI	Moderate
Taylor et al. 2012	Community survey	Hoy/JBI	Moderate
Liu et al. 2016	Programme analysis	ROBINS‐I	Moderate

Abbreviations: JBI, Joanna Briggs Institute; ROBINS‐I, Risk of Bias In Non‐randomised Studies of Interventions.

### 3.3 Pterygium

Two studies assessed pterygium within Indigenous Australians. Both papers included populations of clinic attendees. Landers and Henderson [5] conducted a cross‐sectional survey of exclusively Indigenous patients presenting to very remote community eye clinics within the Northern Territory between 2005 and 2008 for routine check‐ups. The study included 1, 884 patients aged 20+ years in their analysis. Patients were assessed by consultant Ophthalmologists, noting the presence of pterygium at the slit lamp. Visual acuity and subjective refraction were also performed by optometrists. Landers and Henderson found that 7.8% of patients had either pterygium or prior pterygium surgery in either eye. Patients with more advanced age had higher prevalence of pterygium; those aged 40+ years with 9.3%, and 50+ years with 10.6%. There was no significant difference in laterality of the affected eye (5.2% RE, 5.9% LE, *x* ^2^ = 0.19; *p* = 0.91), or the gender of patients with pterygium (*x* ^2^ = 1.06; *p* = 0.3). Visual acuity was also not significantly different between affected and unaffected patients (6/7.5 vs. 6/7, respectively), for right eyes, *t* = 1.62; *p* = 0.11 and 6/7.3 vs. 6/7, respectively, for left eyes, *t* = 1.20; (*p* = 0.23).

McGlacken‐Byrne et al. [6] recorded the presence of pterygium in a retrospective audit of patients who presented to the Lions Outback Vision Visiting Optometry Service in 2017. Individual records of 2072 patients were reviewed, with pterygium present in 293 patients (14.1%). 1, 754 patients identified as Aboriginal (84.7%). Pterygium was present in 14.8% of Indigenous, and 10.8% of non‐Indigenous participants. Non‐Aboriginal males had a higher rate than non‐Aboriginal females (18.0% vs. 6.4%), whereas Aboriginal males had a lower rate than Aboriginal females (11.7% vs. 17%). The authors state a medial visual acuity of 6/6 in all patients with pterygium but did not provide any comment on visual acuity difference between groups.

### 3.4 Keratitis

Two studies reporting keratitis affecting Indigenous Australians were identified, both being retrospective cohort studies based at the Royal Darwin Hospital, NT. Richards et al. [7] included all hospitalised microbial keratitis cases between 2007 and 2014 with 111 patients admitted with keratitis, 23.4% of which were Indigenous. Characteristics and risk factors among the included patients were examined, notable differences included contact lens use in 3.85% of Indigenous vs. 65.88% in non‐Indigenous cases (*p* < 0.0001). Trauma was associated with 50% of Indigenous, and 16.47% of non‐Indigenous cases (*p* = 0.0012). 30.77% of Indigenous patients discharged against medical advice, compared to 2.35% in the non‐Indigenous (*p* = 0.0001).

Kim et al. examined fungal keratitis cases from 2014 to 2022 treated at Royal Darwin Hospital [8]. In this study, 31 patients were identified from review of culture results. Descriptive statistics were used to group eyes into good, moderate and poor outcomes based upon final visual acuity and if any surgical intervention was required. The authors did not provide final outcomes by Indigenous status. Of the entire patient cohort, 26% of patients were Aboriginal or Torres Strait Islander. No Indigenous patients were contact‐lens wearers, whereas 58% of the non‐Indigenous were (*p* = 0.04). Ocular trauma was associated with 38% of cases in the Indigenous group, and 29% in the non‐Indigenous (*p* = 0.04). No risk factor was identified in 28% of Indigenous cases, and 25% were ‘other’, which included working with or playing in dirt (*p* = 0.04). Indigenous patients were younger, with a median of 28 years of age, and 42 years of age in the non‐Indigenous (*p* = 0.047). Indigenous patients presented later from onset of symptoms with a median of 6 days (IQR (interquartile range) 4.5–7.5), compared with 2 days for non‐Indigenous (IQR 1–5.5). The authors posited that the frequently ‘remote' and ‘very remote' living status of Indigenous patients contributed to this disparity.

### 3.5 Trachoma and Trichiasis

Eighteen publications reported on trachoma and/or trichiasis affecting Indigenous Australians. Several publications were based upon data from larger surveys, including the recurring Australian Trachoma Surveillance Annual Report from 2010 to 2022 [9, 10, 11, 12, 13], and the National Indigenous Eye Health Survey [14, 15, 16], and the Central Australian Ocular Health Study [17, 18, 19]. Most studies focused on remote or very remote communities in the Northern Territory, Western Australia, and South Australia. Active trachoma prevalence was primarily studied in Indigenous Children, whereas trichiasis was almost exclusively reported among adults. Study methodologies varied widely and included surveillance reports, community‐based surveys, cross‐sectional studies, and one review.

### 3.6 Active Trachoma Prevalence

Endemic levels of trachoma in Indigenous Children within at‐risk communities were consistently found across national surveillance reports from 2010 to 2014. Follicular Trachoma (TF) prevalence within this period ranged from 0% to 17% depending on locality. Active trachoma prevalence appeared to generally decline from 2010 onwards [9, 10, 11, 12, 13, 19, 20, 21]. The Northern Territory showed the highest burden of trachoma. The 2014–2022 surveillance report update [10] showed a progressive reduction in TF prevalence across most jurisdictions, with many communities falling below the 5% elimination threshold by the late 2010s. Despite this, pockets of higher prevalence persisted in some remote communities, particularly those with lower screening or treatment coverage.

Population‐based surveys found much lower overall rates of trachoma—Taylor et al. [14] found an estimated TF prevalence of approximately 2.8% among Indigenous children within the National Indigenous Eye Health Survey. TF prevalence ranged from 0% to 23%, demonstrating significant regional heterogeneity.

Across the Australian Trachoma Surveillance Annual Reports, communities classified as ‘at risk’ were identified through prior detection of trachoma and jurisdictional public health assessment and represented remote communities requiring ongoing surveillance rather than a population‐based sample. These communities formed the basis of all prevalence estimates and statistics. The number of at‐risk communities varied over time, with 150 communities included in 2010, increasing slightly to 152 in 2011 and peaking at 195 in 2012, before declining to 183 in 2013 [9, 11, 12, 13]. In the surveillance update covering 2014–2022, the number of at‐risk communities declined further, with 125 communities identified as at risk in 2014 and 79 communities in 2022 [10]. Across all reporting periods, at‐risk communities were predominantly located in the Northern Territory, Western Australia, and South Australia.

A significant decline in active trachoma was observed at the community level. Taylor et al. [22] recorded the rate of active trachoma within a single remote community across two points in 1975 and then in 2007 in the Katherine Region of the Northern Territory. Clinical photography and examination were used to detect the presence of TF in children aged 5–13 years. Eighty‐two children were assessed in 1975 and 92 in 2007. The prevalence of active trachoma in the children was 59% and 23% respectively, demonstrating a significant reduction in one community, but not beyond endemic levels. The researchers attribute improvements in housing, sanitation, and access to antibiotic therapies as possible factors for the improvement in trachoma prevalence over this period.

Two studies incorporated laboratory confirmation with clinical examination [20, 21]. Lynch et al. reported discrepancies between clinically graded TF and polymerase chain reaction (PCR)‐confirmed *Chlamydia trachomatis* infection within Indigenous children. The researchers found a high level of discordance between rates of active trachoma identified clinically as opposed to PCR testing. Their study consisted of three cross‐sectional screening surveys between 2019 and 2021 within a small community in northwest Queensland. TF was found in 7% of examined children overall, but only 0.7% of children were PCR‐positive for *C. trachomatis* [20] Lynch et al. replicated these findings in their screening programme for trachoma in the Torres Strait Islands between 2016 and 2019 [21]. In this programme, 7% of Torres Strait children aged 5–9 years showed signs of active follicular inflammation, but only 0.7% of children had PCR‐positivity for C. trachomatous via conjunctival swab. The researchers also performed bacterial culture from conjunctival swabs, with a culture‐positive rate of 35%, growing predominantly *H. influenzae* and *S. aureus* . Anti‐ *C. trachomatis* serology testing (anti‐Pgp3) was done in children aged 1–9 years, with seropositivities of 13% in 2019 and 7% in 2021. The authors note that in Western Pacific communities with endemic trachoma, Pgp3 seroprevalence is typically between 35% and 59% (Table 3).

**TABLE 3:** Follicular trachoma rates of screened Indigenous children aged 1–14 years.

Year	Data source	Prevalence estimate (%)	WA	SA	NT	NSW	Screening coverage (%)
2010	TNIEHS	2.8					
2010	ATSAR	11	9	17	12		63
2011	ATSAR	6	6	3	6		48
2012	ATSAR	3.3	4	1	4		58
2013	ATSAR	4	3.8	3.5	5	0.5	84
2014	ATSAR	4.3	3.6	2.8	5.8	0	89
2022	ATSAR	2	2.9	0	2.1		91

*Note:* Grey shading indicates data not reported. Age ranges reflect those reported in the original surveillance datasets.

Abbreviations: ATSAR, Australian Trachoma Surveillance Annual Report; NSW, New South Wales; NT, Northern Territory; SA, South Australia; TNIEHS, The National Indigenous Eye Health Survey; WA, Western Australia.

### 3.7 Trachomatous Trichiasis Prevalence

Eight studies reported on the prevalence of trachomatous trichiasis [9, 10, 11, 12, 13, 14, 17, 23], usually as a secondary endpoint alongside trachoma prevalence. For instance, in the National Trachoma Surveillance Annual Report (2010–2024), adults were opportunistically examined alongside children. While these reports generally found a low overall prevalence, significantly higher trichiasis rates were observed in certain states and territories. For example, an overall prevalence of 2.12% was observed within the Australian Trachoma Surveillance Report, 2010 [9], compared to a state‐wide prevalence of 10% in Western Australia during the same period. Higher prevalence at the state‐level was also seen in Landers et al. [17] analysis of the Central Australian Ocular Health Study, whereby a rate of 6.1% was observed within the Northern Territory. While regional heterogeneity in trichiasis prevalence can be seen in the early to mid‐2010s, more recent national sampling has yielded generally lower prevalence estimates across all localities. Dirani et al. [23] observed a trichiasis rate of 0.17% among Indigenous adults across 30 geographic areas, spanning five states, and the most recent National Trachoma Surveillance Annual Report [10] estimated an overall prevalence of 0.08% in 2022, with the highest local prevalence being 0.2% in Western Australia over the study period (Table 4).

**TABLE 4:** Trachomatous Trichiasis prevalence among screened Indigenous adults (≥ 40 years: TNIEHS, ATSAR; ≥ 20 years: TCAOHS).

Year	Data source	Prevalence estimate (%)	WA	SA	NT	NSW	Screening coverage (%)
2010	TCAOHS	6.1					
2010	TNIEHS	1.4					
2010	ATSAR	2.12	10	2	6		8
2011	ATSAR	1.61	1	1	4		9
2012	ATSAR	2.1	1	1	5		3
2013	ATSAR	1.27	0.5	0.6	4		
2014	ATSAR	0.5					
2018	TNIEHS	0.17					
2022	ATSAR	0.08	0.2	0.1	0		

*Note:* Grey shading indicates data not reported. Screening coverage reported where available.

Abbreviations: ATSAR, Australian Trachoma Surveillance Annual Report; NT, Northern Territory; SA, South Australia; TCAOHS, The Central Australian Ocular Health Study; TNIEHS, The National Indigenous Eye Health Survey; WA, Western Australia; NSW, New South Wales.

### 3.8 Trachoma, Trichiasis, and Corneal‐Related Vision Loss

Three studies assessed corneal sequelae and vision loss attributable to trachoma and trichiasis. Taylor et al. [16] found the presence of tarsal conjunctival scarring in 15.7% of adults within The National Indigenous Eye Health Survey Cohort. Corneal opacity was seen in 0.3% of participants. In a separate study of the same cohort, Taylor et al. [14] attributed rates of bilateral low vision (VA < 6/12 to ≥ 6/60) or blindness (VA < 6/60) in Indigenous children and adults due to trachoma and its complications. They found that 0.25% of adults in the cohort had bilateral low vision, and 0.17% had bilateral blindness. No children in the cohort had either bilateral low vision or blindness due to trachoma and its sequelae. A third study by Landers et al. [18], derived from the Central Australian Ocular Health Study, examined bilateral low vision and blindness in adults aged 20 years or more within the Northern Territory, Central Australian Statistical Local Area (very remote). Of those with bilateral visual impairment, 2.2% were due to trachoma and its complications, and 13.2% of those with bilateral blindness.

### 3.9 Trachoma Intervention

Three prevalence studies included data and evaluation of treatment delivery and community intervention strategies—Liu et al. [24], Lansingh et al. [25] and the recurring National Trachoma Surveillance Annual Report 2010–2022 [9, 10, 11, 12, 13]. The national surveillance studies provided figures on the administration of azithromycin for active cases of trachoma, household and community contacts each year. In 2010, the delivery of antibiotics was low at 57% overall, due to only 41% of active cases receiving treatment in the Northern Territory [25]. In the same period, 98% of cases in Western Australia received treatment. In 2011, the percentage of active trachoma cases and contacts receiving treatment improved dramatically, with 88% overall, and 88% of cases in the Northern Territory receiving treatment. In subsequent years, treatment coverage remained in the 90% range overall. Detailed azithromycin treatment coverage data were available from national surveillance reports between 2010 and 2013; later surveillance updates primarily reported prevalence and community classification outcomes, with treatment delivery described descriptively rather than as extractable coverage estimates.

Liu et al. examined the impact of various community drug administration (azithromycin) strategies and their effect on active trachoma prevalence in children aged 5–9 years [24]. For their analysis, 182 Aboriginal communities at risk for trachoma were included. Communities were assigned to either no treatment, active case and household contact only treatment, and community‐wide treatment. The authors estimated the change in trachoma prevalence between sequential pairs of years and across multiple years according to treatment strategy using random‐effects meta‐analyses. Only communities with trachoma levels below the 5% endemic threshold could be chosen to not receive treatment. The researchers found the greatest absolute prevalence reduction prevalence estimates of −14% (−0.20 to −0.09 95% CI) following community‐wide azithromycin. Communities that were treated at least once but not with a community‐wide strategy (i.e., active cases and household contacts only) saw no significant prevalence change (0%, −0.03% to +0.03% 95% CI). Communities receiving no treatment saw a small increase of +0.01% (−0.03% to 0.04% 95% CI).

Lansingh et al. [25] assessed the efficacy of environmental changes (‘E' as per the WHO's ‘SAFE' strategy). Two Aboriginal communities of comparable population within Central Australia were selected. Both populations received community‐wide azithromycin plus health and facial cleanliness promotion. One community also received housing and environmental improvements. Active trachoma prevalence declined significantly from baseline to 12 months in both communities (*χ* ^2^ test, *p* < 0.01), with no clear difference in effectiveness between the two.

## 4 Discussion

This review synthesised contemporary evidence on corneal disease affecting Aboriginal and Torres Strait Islander peoples, spanning population surveys, trachoma surveillance, clinic‐based audits, and programme evaluations from the last 15 years. Across conditions, a consistent theme was that corneal morbidity in Indigenous Australians is shaped by structural factors such as geography, service access, continuity of care, and environmental exposure, in addition to biological disease mechanisms. The evidence base is dominated by trachoma, reflecting both its public health priority and the relative maturity of surveillance infrastructure compared with other corneal diseases.

Trachoma remains the clearest example of both progress and ongoing vulnerability for Aboriginal and Torres Strait Islander peoples. Australia continues to stand out internationally as a high‐income nation where trachoma has persisted in remote Indigenous communities, despite longstanding global elimination commitments and established frameworks [3]. The national surveillance series documents meaningful gains over the last decade, with reductions in the number of communities classified as ‘at‐risk’ and reported prevalence in children falling below the 5% endemicity threshold across jurisdictions by 2022 [26]. These findings align with broader understandings that sustained antibiotic delivery, screening, and community‐level public health measures can reduce active disease transmission and limit late‐stage sequelae [25, 27, 28]. Importantly, surveillance data also highlight that progress has not been uniform; pockets of higher prevalence have persisted, particularly where screening and treatment coverage are lower [10].

These contemporary findings should be interpreted against a longer historical backdrop. The National Trachoma and Eye Health Program and Taylor's 1980 reports from northwestern Australia documented very high burdens of corneal opacity, pterygium, and climatic droplet keratopathy among Aboriginal communities, underscoring that ocular surface disease in remote settings has long been shaped by environmental exposure and inequitable access to care [29, 30, 31]. Although these landmark studies pre‐date our pre‐specified search window and were not included in the formal synthesis, they broaden the relevance of the present review by showing both the longstanding nature of this burden and the degree to which recent trachoma gains sit within a much longer history of corneal disease inequity.

Trachomatous trichiasis (TT) prevalence has declined in more recent datasets, consistent with reduced transmission and improved trachoma control [10, 23]. Nevertheless, trichiasis remains an important marker of historical disease burden and ongoing risk among older adults in some communities [12, 17, 18]. Population‐based estimates from the National Eye Health Survey indicate very low overall prevalence, but still higher rates than among non‐Indigenous Australians [23]. Even at low prevalence, trichiasis requires durable systems for detection, referral, surgical management, and long‐term follow‐up, which can be difficult to sustain in very remote settings reliant on outreach‐based ophthalmic services.

Evidence regarding treatment strategies was strongest for antibiotic‐focused interventions delivered at scale. National surveillance reports demonstrate progressive improvements in treatment coverage over time, with most jurisdictions achieving high proportions of cases and contacts treated in later years [9, 10, 11, 12, 13]. Analytic work by Liu et al. demonstrated the greatest reductions in trachoma prevalence following community‐wide azithromycin distribution, whereas strategies limited to active cases and household contacts did not result in significant change [24]. Lansingh et al. evaluated environmental and housing improvements alongside antibiotic administration and health promotion, reporting significant reductions in both communities without clear evidence that one SAFE‐based strategy was superior to the other [25]. These findings were consistent with the effectiveness of antibiotic delivery in reducing active disease but also highlight the difficulty of demonstrating incremental benefit of individual SAFE components in small, community‐level studies.

In contrast, the evidence base for non‐trachomatous corneal disease remains sparse and is largely derived from clinic‐attending populations. For pterygium, only two studies were identified, both limited by sampling frame. In the Central Australian clinic‐based cohort reported by Landers et al., the prevalence of pterygium or prior pterygium surgery among Indigenous adults was 7.8%, with confidence intervals reported for the sampled clinic population, although generalisability was limited [5]. In contrast, confidence intervals were not consistently reported in the optometry service audit by McGlacken‐Byrne et al. [6], limiting formal comparison between Indigenous and non‐Indigenous groups and precluding pooled estimation. The absence of population‐based prevalence estimates with confidence intervals stratified by Indigenous status remains a major barrier to accurately quantifying disease burden and associated uncertainty.

For keratitis, the available evidence suggests not only differences in outcomes but also distinct risk factor profiles between Indigenous and non‐Indigenous patients. In keratitis cohorts from the Top End of the Northern Territory, Indigenous patients had worse final visual acuity despite similar presenting severity, with higher rates of discharge against medical advice and loss to follow‐up [7, 8]. Richards et al. and Kim et al. similarly demonstrated differences in exposure patterns, with Indigenous patients presenting at a younger age and later in the disease course. Importantly, both studies highlighted stark contrasts in risk factors: preceding contact lens wear, a dominant risk factor in urban Australian keratitis, was rare among Indigenous patients, whereas ocular trauma was substantially more common. These findings suggest that keratitis in Indigenous Australians is driven less by lifestyle‐related risk and more by environmental and occupational exposures, compounded by geographic barriers to timely care. These findings have implications for prevention strategies, which should prioritise injury prevention, early recognition, and rapid referral pathways rather than relying on paradigms derived primarily from contact lens‐associated disease.

Several limitations of the evidence base warrant consideration. Heterogeneity in sampling frames, case definitions, and outcome reporting limited comparability and precluded meta‐analysis. Many studies did not consistently report Indigenous identification methods, follow‐up completeness, or patient‐reported outcomes, constraining interpretation of equity and lived experience. Evidence relating to microbial keratitis was derived predominantly from a single geographic region in northern Australia, which may limit generalisability to other settings. In addition, several large Australian keratitis cohorts were not eligible for inclusion because Indigenous status was not reported or analyses were not stratified, highlighting a broader gap in equity‐focused data reporting. Finally, the absence of eligible studies for several corneal pathologies likely reflects both true evidence gaps and methodological challenges in studying uncommon conditions in remote settings.

While trachoma control efforts have yielded substantial progress, persistent inequities and major data gaps remain, particularly for non‐trachomatous corneal disease. Addressing these gaps will require improved Indigenous‐specific surveillance, culturally safe engagement, and better integration of eye health within primary care and outreach models to support timely detection, treatment, and continuity of care.

## Funding

The authors have nothing to report.

## Conflicts of Interest

The authors declare no conflicts of interest.

## Supporting information


**Appendix A:** PRISMA 2020 flow diagram.
Supplement 1—Detailed search strategies.
Supplement 2 –Risk of bias assessment.
**Table S1:** Domain‐level risk of bias assessment of prevalence‐focussed studies (Hoy et al./Joanna Briggs Institute tool). Notes: ‘Moderate' ratings were driven primarily by selection bias and incomplete reporting of participation rates, not outcome measurement. All studies used standardised clinical definitions, resulting in low measurement bias.
**Table S2:** Domain‐level risk of bias assessment of non‐randomised studies (Risk of Bias In Non‐randomised Studies—of Interventions ‘ROBINS‐I’). Notes: no study reached ‘serious’ or ‘critical’ risk of bias. Confounding was unavoidable due to observational design and lack of control groups, but outcome ascertainment was robust.
Appendix B.—Pterygium.
**Table S3:** Pterygium studies and key data. Adapted from Landers J, Henderson T, Craig J. Clinical & Experimental Ophthalmology. 2011;39:604–606, and McGlacken‐Byrne AB, Drinkwater JJ, Mackey DA, Turner AW. Clinical & Experimental Optometry. 2021;104:74–77 (22,23).
Appendix C.—Keratitis.
**Table S4:** Keratitis studies and key data. Adapted from Richards AD, Stewart CM, Karthik H, Petsoglou C. Clinical & Experimental Ophthalmology. 2016;44:205–207, and Kim LN, Karthik H, Proudmore KE, Kidd SE, Baird RW. Tropical Medicine and Infectious Disease. 2024;9 (24,25).
Appendix D.—Trachoma and trichiasis prevalence.
**Table S5:** Trachoma and trichiasis rates reported within Australian Trachoma Surveillance Annual Reports. Adapted from the Australian Trachoma Surveillance Annual Reports (2010–2013) and the Australian Trachoma Surveillance Report update (2014–2022), published in Communicable Diseases Intelligence (8,10,18,19,26).
**Table S6:** Trachoma and trichiasis statistics from studies using National Indigenous Eye Health Survey data. Adapted from Taylor HR, et al. Medical Journal of Australia. 2010;192:248–253 and 312–318, and Dirani M, et al. Clinical & Experimental Ophthalmology. 2018;46:13–17 (27,28,11).
**Table S7:** Characteristics of miscellaneous trachoma prevalence studies not included in national surveillance or large population‐based surveys.
Appendix E.—trachoma control strategies.
**Table S8:** Community drug administration strategies and change in active trachoma prevalence. Adapted from Liu L, et al. PLoS Negl Trop Dis 2016;10:e0004921, and Lansingh VC, et al. Int Ophthalmol 2010;30:489–497.
**Table S9:** Azithromycin treatment coverage among children aged 1–14 years with trachoma, derived from Australian Trachoma Surveillance Annual Reports (2010–2013).
Appendix F.—Non‐trachomatous corneal vision loss.
**Table S10:** Vision loss prevalence not attributable to trachoma or trichiasis. Adapted from Landers J, et al. British Journal of Ophthalmology. 2010; 94:1140–1144, and Foreman J, et al. JAMA Ophthalmology. 2018; 136:240–248 (30, 29).

## Data Availability

The data that supports the findings of this study are available in the Supporting Information of this article.

## References

[1] S. R. Flaxman , R. R. A. Bourne , S. Resnikoff , et al., “Global Causes of Blindness and Distance Vision Impairment 1990–2020: A Systematic Review and Meta‐Analysis,” Lancet Global Health 5, no. 12 (2017): e1221–e1234. 29032195 10.1016/S2214-109X(17)30393-5

[2] S. Resnikoff , D. Pascolini , D. Etya'ale , et al., “Global Data on Visual Impairment in the Year 2002,” Bulletin of the World Health Organization 82, no. 11 (2004): 844–851. 15640920 PMC2623053

[3] Organisation WH , “Global Elimination of Blinding Trachoma,” 2013, https://www.who.int/publications/i/item/wha51.11.

[4] J. Foreman , J. Xie , S. Keel , et al., “The Prevalence and Causes of Vision Loss in Indigenous and Non‐Indigenous Australians: The National Eye Health Survey,” Ophthalmology 124, no. 12 (2017): 1743–1752. 28689897 10.1016/j.ophtha.2017.06.001

[5] J. Landers , T. Henderson , and J. Craig , “Prevalence of Pterygium in Indigenous Australians Within Central Australia: The Central Australian Ocular Health Study,” Clinical & Experimental Ophthalmology 39, no. 7 (2011): 604–606. 22452680 10.1111/j.1442-9071.2011.02532.x

[6] A. B. McGlacken‐Byrne , J. J. Drinkwater , D. A. Mackey , and A. W. Turner , “Gender and Ethnic Differences in Pterygium Prevalence: An Audit of Remote Australian Clinics,” Clinical & Experimental Optometry 104, no. 1 (2021): 74–77. 32363676 10.1111/cxo.13081

[7] A. D. Richards , C. M. Stewart , H. Karthik , and C. Petsoglou , “Microbial Keratitis in Indigenous Australians,” Clinical & Experimental Ophthalmology 44, no. 3 (2016): 205–207. 26350024 10.1111/ceo.12643

[8] L. N. Kim , H. Karthik , K. E. Proudmore , S. E. Kidd , and R. W. Baird , “Fungal Keratitis, Epidemiology and Outcomes in a Tropical Australian Setting,” Tropical Medicine and Infectious Disease 9, no. 6 (2024): 127. 38922039 10.3390/tropicalmed9060127 PMC11209044

[9] C. S. Cowling , G. Povovic , B. C. Liu , et al., “Australian Trachoma Surveillance Annual Report, 2010,” Communicable Diseases Intelligence Quarterly Report 36, no. 3 (2012): E242‐50. 23186235 10.33321/cdi.2012.36.18

[10] A. Jaworski , C. Cowling , G. C. Popovic , et al., “Australian Trachoma Surveillance Report Update: 2014–2022,” Communicable Diseases Intelligence 49 (2025): 3–16. 10.33321/cdi.2025.49.006 39837616

[11] C. S. Cowling , B. C. Liu , J. Ward , T. L. Snelling , J. M. Kaldor , and D. Wilson , “Australian Trachoma Surveillance Annual Report, 2011,” Communicable Diseases Intelligence Quarterly Report 37, no. 2 (2013): E121–E129. 24168085 10.33321/cdi.2013.37.17

[12] C. S. Cowling , B. C. Liu , T. L. Snelling , J. S. Ward , J. M. Kaldor , and D. P. Wilson , “National Trachoma Surveillance Annual Report, 2012,” Communicable Diseases Intelligence Quarterly Report 39, no. 1 (2015): E146–E157. 26063088 10.33321/cdi.2015.39.9

[13] C. S. Cowling , B. C. Liu , T. L. Snelling , J. S. Ward , J. M. Kaldor , and D. P. Wilson , “Australian Trachoma Surveillance Annual Report, 2013,” Communicable Diseases Intelligence Quarterly Report 40, no. 2 (2016): E255–E266. 27522137 10.33321/cdi.2016.40.21

[14] H. R. Taylor , S. S. Fox , J. Xie , R. A. Dunn , A. L. Arnold , and J. E. Keeffe , “The Prevalence of Trachoma in Australia: The National Indigenous Eye Health Survey,” Medical Journal of Australia 192, no. 5 (2010): 248–253. 20201757 10.5694/j.1326-5377.2010.tb03501.x

[15] J. Foreman , J. Xie , S. Keel , et al., “Prevalence and Causes of Unilateral Vision Impairment and Unilateral Blindness in Australia: The National Eye Health Survey,” JAMA Ophthalmology 136, no. 3 (2018): 240–248. 29372249 10.1001/jamaophthalmol.2017.6457 PMC5885895

[16] H. R. Taylor , J. Xie , S. Fox , R. A. Dunn , A. L. Arnold , and J. E. Keeffe , “The Prevalence and Causes of Vision Loss in Indigenous Australians: The National Indigenous Eye Health Survey,” Medical Journal of Australia 192, no. 6 (2010): 312–318. 20230347 10.5694/j.1326-5377.2010.tb03529.x

[17] J. Landers , T. Henderson , and J. Craig , “Prevalence and Associations of Blinding Trachoma in Indigenous Australians Within Central Australia: The Central Australian Ocular Health Study,” Clinical & Experimental Ophthalmology 38, no. 4 (2010): 398–404. 20665942 10.1111/j.1442-9071.2010.02288.x

[18] J. Landers , T. Henderson , and J. Craig , “The Prevalence and Causes of Visual Impairment in Indigenous Australians Within Central Australia: The Central Australian Ocular Health Study,” British Journal of Ophthalmology 94, no. 9 (2010): 1140–1144. 20530181 10.1136/bjo.2009.168146

[19] J. Landers , T. Henderson , and J. E. Craig , “Incidence of Visual Impairment due to Cataract, Diabetic Retinopathy and Trachoma in Indigenous Australians Within Central Australia: The Central Australian Ocular Health Study,” Clinical & Experimental Ophthalmology 41, no. 1 (2013): 50–55. 22594763 10.1111/j.1442-9071.2012.02817.x

[20] K. D. Lynch , W. Morotti , G. Brian , et al., “Clinical Signs of Trachoma and Laboratory Evidence of Ocular *Chlamydia trachomatis* Infection in a Remote Queensland Community: A Serial Cross‐Sectional Study,” Medical Journal of Australia 217, no. 10 (2022): 538–543. 36180097 10.5694/mja2.51735 PMC9827872

[21] K. D. Lynch , G. Brian , T. Ahwang , et al., “Assessing the Prevalence of Trachoma: Lessons From Community Screening With Laboratory Testing in Australia's Torres Strait Islands,” Ophthalmic Epidemiology 30, no. 6 (2023): 663–670. 36281525 10.1080/09286586.2022.2136389

[22] H. R. Taylor , D. R. English , B. A. Field , P. E. Spicer , and D. M. Graham , “Prevalence of Trachoma in a Single Community, 1975–2007,” Clinical & Experimental Ophthalmology 40, no. 2 (2012): 121–126. 21883776 10.1111/j.1442-9071.2011.02672.x

[23] M. Dirani , S. Keel , J. Foreman , P. van Wijngaarden , and H. R. Taylor , “Prevalence of Trachomatous Trichiasis in Australia: The National Eye Health Survey,” Clinical & Experimental Ophthalmology 46, no. 1 (2018): 13–17. 10.1111/ceo.13003 28598533

[24] B. Liu , C. Cowling , A. Hayen , et al., “Relationship Between Community Drug Administration Strategy and Changes in Trachoma Prevalence, 2007 to 2013,” PLoS Neglected Tropical Diseases 10, no. 7 (2016): e0004810. 27385309 10.1371/journal.pntd.0004810 PMC4934776

[25] V. C. Lansingh , B. N. Mukesh , J. E. Keeffe , and H. R. Taylor , “Trachoma Control in Two Central Australian Aboriginal Communities: A Case Study,” International Ophthalmology 30, no. 4 (2010): 367–375. 20358257 10.1007/s10792-010-9360-5

[26] Organisation WH , “Trachoma [Online Database],” 2025, https://www.who.int/news‐room/fact‐sheets/detail/trachoma.

[27] E. Habtamu , E. M. Harding‐Esch , K. Greenland , et al., “Trachoma,” Lancet 405, no. 10492 (2025): 1865–1878. 40412861 10.1016/S0140-6736(25)00551-3 PMC7618282

[28] A. J. Shattock , M. Gambhir , H. R. Taylor , C. S. Cowling , J. M. Kaldor , and D. P. Wilson , “Control of Trachoma in Australia: A Model Based Evaluation of Current Interventions,” PLoS Neglected Tropical Diseases 9, no. 4 (2015): e0003474. 25860143 10.1371/journal.pntd.0003474 PMC4393231

[29] National Trachoma and Eye Health Program of the Royal Australian College of Ophthalmologists (Royal Australian College of Ophthalmologists, 1980).

[30] H. R. Taylor , “The Prevalence of Corneal Disease and Cataracts in Australian Aborigines in Northwestern Australia,” Australian Journal of Ophthalmology 8, no. 4 (1980): 289–301. 7224984 10.1111/j.1442-9071.1980.tb00285.x

[31] H. R. Taylor , “Aetiology of Climatic Droplet Keratopathy and Pterygium,” British Journal of Ophthalmology 64, no. 3 (1980): 154–163. 7387947 10.1136/bjo.64.3.154 PMC1039379

