# Supplementary material for: Prevalence, Treatment, and Outcomes of Corneal Disease in Aboriginal and Torres Strait Islander Peoples in Australia: A Systematic Review
Source: Clin Exp Ophthalmol. 2026 Apr 13;54(5):653–63. doi: 10.1111/ceo.70118 (PMC13340474; doi:10.1111/ceo.70118)
Supplement: Supplementary file 1 — Appendix A: PRISMA 2020 flow diagram. Supplement 1—Detailed search strategies. Supplement 2 –Risk of bias assessment. Table S1: Domain‐level risk of bias assessment of prevalence‐focussed studies (Hoy et al./Joanna Briggs Institute tool). Notes: ‘Moderate' ratings were driven primarily by selection bias and incomplete reporting of participation rates, not outcome measurement. All studies used standardised clinical definitions, resulting in low measurement bias. Table S2: Domain‐level risk of bias assessment of non‐randomised studies (Risk of Bias In Non‐randomised Studies—of Interventions ‘ROBINS‐I’). Notes: no study reached ‘serious’ or ‘critical’ risk of bias. Confounding was unavoidable due to observational design and lack of control groups, but outcome ascertainment was robust. Appendix B.—Pterygium. Table S3: Pterygium studies and key data. Adapted from Landers J, Henderson T, Craig J. Clinical & Experimental Ophthalmology. 2011;39:604–606, and McGlacken‐Byrne AB, Drinkwater JJ, Mackey DA, Turner AW. Clinical & Experimental Optometry. 2021;104:74–77 (22,23). Appendix C.—Keratitis. Table S4: Keratitis studies and key data. Adapted from Richards AD, Stewart CM, Karthik H, Petsoglou C. Clinical & Experimental Ophthalmology. 2016;44:205–207, and Kim LN, Karthik H, Proudmore KE, Kidd SE, Baird RW. Tropical Medicine and Infectious Disease. 2024;9 (24,25). Appendix D.—Trachoma and trichiasis prevalence. Table S5: Trachoma and trichiasis rates reported within Australian Trachoma Surveillance Annual Reports. Adapted from the Australian Trachoma Surveillance Annual Reports (2010–2013) and the Australian Trachoma Surveillance Report update (2014–2022), published in Communicable Diseases Intelligence (8,10,18,19,26). Table S6: Trachoma and trichiasis statistics from studies using National Indigenous Eye Health Survey data. Adapted from Taylor HR, et al. Medical Journal of Australia. 2010;192:248–253 and 312–318, and Dirani M, et al. Clinical & Experimental Ophthalmology. 2 [file CEO-54-653-s001.docx]

**Supplementary material: Corneal Disease and Vision Impairment in Indigenous Australians: A Systematic Review**

Appendix A

## PRISMA 2020 flow diagram

**Identification of studies via databases and registers**

Records removed *before screening*:

Duplicate records removed (n = 906)

Records marked as ineligible by automation tools (n = 0)

Records removed for other reasons (n = 0)

Records identified from*:

Databases (n = 2,943)

Registers (n=0)

**Identification**

Records screened

(n = 2,037)

Records excluded**

(n = 1,983)

Reports sought for retrieval

(n = 54)

Reports not retrieved

(n = 0)

**Screening**

Reports assessed for eligibility

(n = 54)

Reports excluded:

Reason 1 (n = 1)

Reason 2 (n = 2)

Reason 3 (n = 11)

Reason 4 (n = 17)

Reason 5 (n = 2)

Studies included in review

(n = 14)

Reports of included studies

(n = 21)

**Included**

Reason 1 – non-Australian population

Reason 2 – non-Indigenous population only

Reason 3 – not corneal disease

Reason 4 – commentary/editorial/review

Reason 5 – laboratory-only study

## Supplement 1 – detailed search strategies

**PubMed** (685 results)

("Indigenous Peoples"[MeSH] OR "Aboriginal Australian*"[Title/Abstract] OR "Indigenous Australian*"[Title/Abstract] OR "Torres Strait Islanders"[Title/Abstract] OR "First Nations Australians"[Title/Abstract] OR "Native Australians"[Title/Abstract] OR "Native Hawaiian or Other Pacific Islander"[MeSH] OR "Australia"[MeSH] OR "Australian"[Title/Abstract])

AND

("Cornea"[MeSH] OR "Corneal Diseases"[MeSH] OR "Keratitis"[MeSH] OR "Corneal Ulcer*"[MeSH] OR "Corneal Opaci*"[MeSH] OR "Keratocon*"[MeSH] OR "Corneal health"[Title/Abstract] OR "Corneal infection"[Title/Abstract] OR "Corneal scarring"[Title/Abstract] OR "Corneal transplantation"[Title/Abstract] OR "Eye health"[Title/Abstract] OR "Ocular surface disease"[Title/Abstract] OR "Keratopathy"[Title/Abstract] OR "Corneal dystrophy"[Title/Abstract] OR "Pterygium"[MeSH] OR "Trachoma"[MeSH] OR "Dry Eye Syndromes"[MeSH] OR "Keratitis" [Title/Abstract])

AND

"Humans" [Mesh]

**Scopus** (1,046 results)

( TITLE-ABS-KEY ( "Indigenous Peoples" ) OR TITLE-ABS-KEY ( "Aboriginal Australian*" ) OR TITLE-ABS-KEY ( "Indigenous Australian*" ) OR TITLE-ABS-KEY ( "Torres Strait Islanders" ) OR TITLE-ABS-KEY ( "First Nations Australians" ) OR TITLE-ABS-KEY ( "Native Australians" ) OR TITLE-ABS-KEY ( "Native Hawaiian or Other Pacific Islander" ) OR TITLE-ABS-KEY ( "Australia" ) OR TITLE-ABS-KEY ( "Australian" ) ) AND ( TITLE-ABS-KEY ( "Cornea" ) OR TITLE-ABS-KEY ( "Corneal Diseases" ) OR TITLE-ABS-KEY ( "Keratitis" ) OR TITLE-ABS-KEY ( "Corneal Ulcer*" ) OR TITLE-ABS-KEY ( "Corneal Opaci*" ) OR TITLE-ABS-KEY ( "Keratocon*" ) OR TITLE-ABS-KEY ( "Corneal health" ) OR TITLE-ABS-KEY ( "Corneal infection" ) OR TITLE-ABS-KEY ( "Corneal scarring" ) OR TITLE-ABS-KEY ( "Corneal transplantation" ) OR TITLE-ABS-KEY ( "Eye health" ) OR TITLE-ABS-KEY ( "Ocular surface disease" ) OR TITLE-ABS-KEY ( "Keratopathy" ) OR TITLE-ABS-KEY ( "Corneal dystrophy" ) OR TITLE-ABS-KEY ( "Pterygium" ) OR TITLE-ABS-KEY ( "Trachoma" ) OR TITLE-ABS-KEY ( "Dry Eye Syndromes" ) OR TITLE-ABS-KEY ( "Keratitis" ) ) AND ( LIMIT-TO ( AFFILCOUNTRY , "Australia" ) )

+ Australia regional filter applied

**Embase** (1,194 results)

('indigenous people' OR 'aboriginal australian*':ti,ab OR 'indigenous australian*':ti,ab OR 'torres strait islander*':ti,ab OR 'first nations australian*':ti,ab OR 'native australian*':ti,ab OR 'native hawaiian or other pacific islander' OR 'australia' OR 'australian*':ti,ab)

AND

('cornea' OR 'corneal disease' OR 'keratitis' OR 'corneal ulcer' OR 'corneal opacification' OR 'keratoconus' OR 'corneal health':ti,ab OR 'corneal infection':ti,ab OR 'corneal scarring':ti,ab OR 'corneal transplantation':ti,ab OR 'eye health':ti,ab OR 'ocular surface disease':ti,ab OR 'keratopathy':ti,ab OR 'corneal dystrophy':ti,ab OR 'pterygium' OR 'trachoma' OR 'dry eye syndrome' OR 'keratitis':ti,ab)

## Supplement 2 –risk of bias assessment

| **Study** | **Sampling frame** | **Representativeness** | **Case definition** | **Measurement** | **Response rate** | **Overall RoB** |
| --- | --- | --- | --- | --- | --- | --- |
| Australian Trachoma Surveillance Program | Moderate | Moderate | Low | Low | Unclear | **Moderate** |
| National Indigenous Eye Health Survey | Low | Low | Low | Low | Low | **Low** |
| National Eye Health Survey | Low | Low | Low | Low | Low | **Low** |
| Central Australian Ocular Health Study | Moderate | Moderate | Low | Low | Unclear | **Moderate** |
| Taylor et al., 2011 (single community) | Moderate | Moderate | Low | Low | Unclear | **Moderate** |
| Lynch et al., 2022 (Torres Strait) | Moderate | Moderate | Low | Low | Unclear | **Moderate** |
| Lynch et al., 2022 (remote QLD) | Moderate | Moderate | Low | Low | Unclear | **Moderate** |
| Landers & Henderson, 2011 (pterygium) | Moderate | Moderate | Low | Low | Unclear | **Moderate** |
| McGlacken-Byrne et al., 2020 | Moderate | Moderate | Low | Low | Unclear | **Moderate** |
| Foreman et al., 2018 | Low | Low | Low | Low | Low | **Low** |

Supplementary Table 1: domain-level risk of bias assessment of prevalence-focussed studies (Hoy et al./Joanna Briggs Institute tool). Notes: ‘Moderate’ ratings were driven primarily by selection bias and incomplete reporting of participation rates, not outcome measurement. All studies used standardised clinical definitions, resulting in low measurement bias

| **Study** | **Confounding** | **Selection** | **Intervention classification** | **Missing data** | **Outcome measurement** | **Reporting** | **Overall RoB** |
| --- | --- | --- | --- | --- | --- | --- | --- |
| Liu et al., 2016 | Moderate | Moderate | Low | Low | Low | Low | **Moderate** |
| Lansingh et al., 2010 | Moderate | Moderate | Low | Low | Low | Low | **Moderate** |
| Richards et al., 2016 | Moderate | Moderate | Low | Moderate | Low | Low | **Moderate** |
| Kim et al., 2024 | Moderate | Moderate | Low | Moderate | Low | Low | **Moderate** |

Supplementary Table 2: domain-level risk of bias assessment of non-randomised studies (Risk of Bias In Non-randomized Studies - of Interventions ‘ROBINS-I’). Notes: no study reached ‘serious’ or ‘critical’ risk of bias. Confounding was unavoidable due to observational design and lack of control groups, but outcome ascertainment was robust.

## Appendix B – Pterygium

| **Design** | **Period** | **Population** | **Location** | **Prevalence (%)** | |
| --- | --- | --- | --- | --- | --- |
| **Prevalence of pterygium in Indigenous Australians within central Australia: The Central Australian Ocular Health Study**  Landers J, Henderson T, Craig J; 2011 (22) | | | | | |
| Clinic-based cross-sectional survey of Indigenous Australians presenting to remote community eye clinics | July 2005 – June 2008 | 1,884 Indigenous Australians aged ≥ 20 yrs | Central Australian Statistical Local Area,  Northern Territory, Australia  (very remote communities) | Overall | 7.8 |
|  |  |  |  | Age ≥ 50 years | 10.6 (95% CI 8.5–12.7) |
|  |  |  |  | Age ≥ 40 years | 9.3 (95% CI 7.7–10.9) |
| **Gender and ethnic differences in pterygium prevalence: an audit of remote Australian clinics**  McGlacken-Byrne A et al., 2020 (23) | | | | | |
| Retrospective clinic database audit of consecutive attendees | Jan-Dec 2017 | 2, 072 clinic attendees, ages (27-90),  84.7% Indigenous | Rural Northern Western Australia outreach clinics:  Kimberley 69.0%,  Pilbara 28.1%,  Goldfields 2.9% of attendees | Overall | 14.1 |
|  |  |  |  | Indigenous | 14.8 |
|  |  |  |  | Non-Indigenous | 10.8 |

Supplementary Table 3: Pterygium studies and key data. Adapted from Adapted from Landers J, Henderson T, Craig J. Clinical & Experimental Ophthalmology. 2011;39:604–606, and McGlacken-Byrne AB, Drinkwater JJ, Mackey DA, Turner AW. Clinical & Experimental Optometry. 2021;104:74–77 (22,23).

## Appendix C – Keratitis

| **Design** | **Period** | **Population** | **Location** | **Key Findings** |
| --- | --- | --- | --- | --- |
| **Microbial Keratitis in Indigenous Australians**  Richards A, Stewart C, Karthik H, Petsoglou C (2015) (24) | | | | |
| Retrospective hospital cohort comparing Indigenous vs non-Indigenous admitted keratitis cases; descriptive statistics with group comparisons | 2007 - 2014 | 111 patients admitted with keratitis, 23.4% Indigenous | Royal Darwin Hospital, NT | **Indigenous** |
|  |  |  |  | CL wear 3.85%  Trauma 50%  Discharge against advice 30.77% |
|  |  |  |  | **Non-Indigenous** |
|  |  |  |  | CL wear 65.88%  Trauma 16.47%  Discharge against advice 2.35% |
| **Fungal Keratitis, Epidemiology and Outcomes in a Tropical Australian Setting**  Kim LN, Karthik H, Proudmore KE, Kidd SE, Baird RW (2024) (25) | | | | |
| Retrospective hospital cohort of culture-proven fungal keratitis with descriptive stats and Fisher’s exact tests. Single centre (Royal Darwin Hospital, NT). | 2014 - 2022 | 31 patients with culture-proven fungal keratitis, 26% Indigenous | Royal Darwin Hospital, NT | **Indigenous** |
|  |  |  |  | CL wear 0%  Trauma 38% |
|  |  |  |  | **Non-Indigenous** |
|  |  |  |  | CL use 58%  Trauma 29% |

Supplementary Table 4: Keratitis studies and key data. Adapted from Richards AD, Stewart CM, Karthik H, Petsoglou C. Clinical & Experimental Ophthalmology. 2016;44:205–207, and Kim LN, Karthik H, Proudmore KE, Kidd SE, Baird RW. Tropical Medicine and Infectious Disease. 2024;9 (24,25).

## Appendix D - Trachoma and trichiasis prevalence

| **Australian Trachoma Surveillance Report (2010 – 2022)** | | | | | | | |
| --- | --- | --- | --- | --- | --- | --- | --- |
| **Design** |  | Cross-sectional surveillance of ‘at-risk’ communities | | | | | |
| **Disease type** |  | Active trachoma, trichiasis | | | | | |
| **Case definition** |  | WHO simplified grading system | | | | | |
| **Prevalence type** |  | Point prevalence (adjusted) | | | | | |
| **Populations** |  | Children 1-14 for trachoma prevalence, adults aged >40 years for trichiasis within ‘at-risk’ communities (>5% TI/TF prevalence in ages 5-9 within last 5 years) | | | | | |
| **Diagnostic tools** |  | Clinical eye examination by trained personnel | | | | | |
| **Population sample** | Overall prevalence estimate (%) | | Screening coverage (%) | WA | SA | NT | NSW |
| **Australian Trachoma Surveillance Annual Report, 2010**  Cowling C et al, 2010 (8) | | | | | | | |
| **Trachoma**  Children aged 11-14 within 150 ‘at-risk’ communities  6,762 Indigenous children aged 1-14 | 11 | | 63 | 9 | 17 | 12 |  |
| **Trichiasis**  1, 036 Indigenous Adults aged 40+ | 2.12 (1.34 – 3.20 95% CI) | | 8 | 10 | 2 | 6 |  |

| **Population sample** | Overall prevalence estimate (%) | Screening coverage (%) | WA | SA | NT | NSW |
| --- | --- | --- | --- | --- | --- | --- |
| **Australian Trachoma Surveillance Annual Report, 2011**  Cowling C et al, 2011 (10) | | | | | | |
| **Trachoma**  152 ‘at-risk’ communities  8, 738 Indigenous children aged 1-14 | 6 | 48 | 6 | 3 | 6 |  |
| **Trichiasis**  1, 179 Indigenous Adults aged 40+ | 1.61 (0.97 – 2.52 95% CI) | 9 | 1 | 1 | 4 |  |
| **Australian Trachoma Surveillance Annual Report, 2012**  Cowling C et al, 2012 (18) | | | | | | |
| **Trachoma**  195 ‘at-risk’ communities  9, 122 Indigenous children aged 1-14 | 3.3 | 58 | 4 | 1 | 4 |  |
| **Trichiasis**  4, 468 Indigenous Adults aged 40+ | 2.1 (1.70 – 2.57 95% CI) | 3 | 1 | 1 | 5 |  |
| **Australian Trachoma Surveillance Annual Report, 2013**  Cowling C et al, 2013 (26) | | | | | | |
| **Trachoma**  183 ‘at-risk’ communities  4, 213 Indigenous children aged 5-9 | 4 | 84 | 3.8 | 3.5 | 5 | 0.5 |
| **Trichiasis**  3, 856 Indigenous aged 15+ | 1.27 (0.94 – 1.68 95% CI) |  | 0.5 | 0.6 | 4 |  |

| **Population sample** | Overall prevalence estimate (%) | Screening coverage (%) | WA | SA | NT | NSW |
| --- | --- | --- | --- | --- | --- | --- |
| **Australian Trachoma Surveillance Report Update 2014 – 2022 (2014 focus)**  Jaworski et al, 2025 (19) | | | | | | |
| **Trachoma**  125 ‘at-risk’ communities  4, 284 Indigenous children aged 5-9 years | 4.3 | 89 | 3.6 | 2.8 | 5.8 | 0 |
| Trichiasis  Adults aged 40+ | 0.5 (no CI) |  |  |  |  |  |
| **Australian Trachoma Surveillance Report Update 2014 – 2022 (2022 focus)**  Jaworski et al, 2025 (19) | | | | | | |
| **Trachoma**  79 ‘at-risk’ communities  1, 491 Indigenous children aged 5-9 years | 2 | 91 | 2.9 | 0 | 2.1 |  |
| **Trichiasis**  Adults aged 40+ | 0.08 (no CI) |  | 0.2 | 0.1 | 0 |  |

*Supplementary table 5: Trachoma and trichiasis rates reported within Australian Trachoma Surveillance Annual Reports. Adapted from the Australian Trachoma Surveillance Annual Reports (2010–2013) and the Australian Trachoma Surveillance Report update (2014–2022), published in Communicable Diseases Intelligence (8,10,18,19,26).*

| **The National Indigenous Eye Health Survey** | | | | | | | |
| --- | --- | --- | --- | --- | --- | --- | --- |
| **Design** | | National, stratified, random-cluster, population-based cross-sectional survey | | | | | |
| **Disease type** | | Trachoma and trichiasis | | | | | |
| **Case definition** | | WHO simplified grading system - active trachoma, tarsal scarring, tarsal inflammation, trichiasis, corneal opacity | | | | | |
| **Prevalence type** | | Point prevalence | | | | | |
| **Populations** | | Indigenous children (5–15 yrs) and adults (≥ 40 yrs) in 30 communities across Australia | | | | | |
| **Diagnostic tools** | | Clinical eye examination by trained personnel | | | | | |
| **Design** | **Period** | | **Location** | **Population** | **Prevalence estimate (%)** | | |
| **The prevalence of trachoma in Australia: The National Indigenous Eye Health Survey**  Taylor HR et al, 2010 (27) | | | | | | | |
| Population-based cross-sectional survey; multi-stage random-cluster sampling, stratified by remoteness | Jan – Dec 2008 | | Australia-wide, 30 sites spanning major city, inner/outer regional, remote, and very remote areas. | 1, 694 Indigenous children aged 5-15 years, 1,189 Indigenous adults aged 40+ as ‘representative sample’ | Children - active trachoma | | 2.8 |
|  |  |  |  |  | Adults -trachomatous trichiasis | | 1.4 |
|  |  |  |  |  | Adults – tarsal conjunctival scarring | | 15.7 |
|  |  |  |  |  | Adults – corneal opacity | | 0.3 |
| **The prevalence and causes of vision loss in Indigenous Australians: The National Indigenous Eye Health Survey**  Taylor HR et al, 2010 (28) | | | | | | | |
| Population-based cross-sectional survey; multi-stage random-cluster sampling, stratified by remoteness | Jan – Dec 2008 | | Australia-wide, 30 sites spanning major city, inner/outer regional, remote, and very remote areas. | 1, 694 Indigenous children aged 5-15 years, 1,189 Indigenous adults aged 40+ as ‘representative sample’ | Bilateral low vision (VA <6/12 to ≥6/60) due to corneal pathology | Children = 0 | |
|  |  |  |  |  |  | Adults = 0.25 | |
|  |  |  |  |  | Bilateral blindness (VA <6/60) due to corneal pathology | Children = 0 | |
|  |  |  |  |  |  | Adults = 0.17 | |

| **Design** | **Period** | **Location** | **Population** | **Prevalence estimate (%)** | |
| --- | --- | --- | --- | --- | --- |
| **Prevalence of trachomatous trichiasis in Australia: The National Eye Health Survey**  Dirani M et al., 2018 (11) | | | | | |
| Population-based cross-sectional survey; multi-stage random-cluster sampling, stratified by remoteness | Mar 2015 – Apr 2016 | Australia-wide, 30 sites spanning major city, inner/outer regional, remote, and very remote areas. | 1,738 Indigenous Australians aged ≥40 years as ‘representative sample’ | Trachomatous trichiasis | 0.17 |

Supplementary table 6: Trachoma and trichiasis statistics from studies using National Indigenous Eye Health Survey data. Adapted from Taylor HR, et al. Medical Journal of Australia. 2010;192:248–253 and 312–318, and Dirani M, et al. Clinical & Experimental Ophthalmology. 2018;46:13–17 (27,28,11).

## Other trachoma prevalence studies

| **Design** | | **Period** | | | | **Location** | | **Population** | | | **Case definition** | | **Prevalence estimate (%)** | | | | | |
| --- | --- | --- | --- | --- | --- | --- | --- | --- | --- | --- | --- | --- | --- | --- | --- | --- | --- | --- |
| **Prevalence of trachoma in a single community, 1975 & 2007**  Taylor et al, 2011 (34) | | | | | | | | | | | | | | | | | | |
| Single-community cross-sectional surveys (population of one community, photo grading).  Point prevalence | | 1975 and 2007 calendar years | | | | Katherine region, Northern Territory | | Children aged 5 – 13 years within same community  N = 82 (1975)  N = 92 (2007) | | | Active trachoma (trachoma follicular ≥3 and or trachoma intense ≥3) as identified by photographs | | 1975 | | | 59 | | |
|  |  |  |  |  |  |  |  |  |  |  |  |  | 2007 | | | 23 | | |
| **Prevalence and associations of blinding trachoma in Indigenous Australians within Central Australia: The Central Australian Ocular Health Study**  Landers J et al., 2010 (30) | | | | | | | | | | | | | | | | | | |
| Prospective population-based longitudinal cohort study  of clinic attendees,  point prevalence | | July 2005 – June 2008 | | | | Central Australian Statistical Local Area, Northern Territory (very remote) | | 1, 884 Indigenous Australians aged ≥ 20 years residing in 30 remote communities who presented to scheduled eye-clinics | | | Trachomatous trichiasis (TT) | | TT | | | 6.1  (5.0-7.2 95% CI) | | |
|  |  |  |  |  |  |  |  |  |  |  | Trachomatous corneal opacification (CO) | | CO | | | 3.3  (2.5-4.1 95% CI) | | |
| **Design** | **Period** | | | | **Location** | | **Population** | | | **Case definition** | | | | | **Prevalence estimate (%)** | | | |
| **Prevalence and causes of visual impairment in Indigenous Australians within Central Australia: The Central Australian Ocular Health Study**  Landers J et al., 2010 (31) | | | | | | | | | | | | | | | | | | |
| Prospective population-based longitudinal cohort study  of clinic attendees,  point prevalence | July 2005 – June 2008 | | | | Central Australian Statistical Local Area, Northern Territory (very remote) | | 1, 884 Indigenous Australians aged ≥ 20 years residing in 30 remote communities who presented to scheduled eye-clinics | | | Bilateral visual impairment (VA < 6/12 OU) | | | | | 19.4 | | | |
|  |  |  |  |  |  |  |  |  |  | Bilateral visual impairment due to trachoma | | | | | 2.2 | | | |
|  |  |  |  |  |  |  |  |  |  | Bilateral blindness (VA <6/60 OU) | | | | | 2.8 | | | |
|  |  |  |  |  |  |  |  |  |  | Bilateral blindness due to trachoma | | | | | 13.2 | | | |
| **Assessing the Prevalence of Trachoma: Lessons from Community Screening with Laboratory Testing in Australia’s Torres Strait Islands**  Lynch KD, Brian G et al, 2022 (36) | | | | | | | | | | | | | | | | | | |
| Serial cross-sectional screening surveys (11 surveys across 7 communities, 4 island clusters)  Point prevalence per-round | | | 2016 – 2019  (Multiple rounds; first surveys aggregated; repeat surveys in some communities | | Torres Strait Islands, Queensland,  across all four outer island groups  PCR testing was negative in all cases | | | | Children aged 5-9 years, adults opportunistically examined  91% Indigenous participants | Trachomatous inflammation - follicular (WHO grading) | | | | | 2016 | | 6 | |
|  |  |  |  |  |  |  |  |  |  |  |  |  |  |  | 2017 | | 3 - 8 | |
|  |  |  |  |  |  |  |  |  |  |  |  |  |  |  | 2018 | | 0 | |
|  |  |  |  |  |  |  |  |  |  |  |  |  |  |  | 2019 | | 4 | |
| **Clinical signs of trachoma and laboratory evidence of ocular Chlamydia trachomatis infection in a remote Queensland community: a serial cross-sectional study**  Lynch KD, Marotti W et al, 2022 (35) | | | | | | | | | | | | | | | | | | |
| Serial cross-sectional community screening (2019, 2020, 2021) with integrated lab testing (PCR done in 2019 for C. trachomatis) | | | 2019 – 2021 (three annual rounds)  PCR testing*** much lower than TF rate and authors recommend no further screening/caution | | One remote north-west Queensland community (population <1500), chosen for ties to NT communities where trachoma persists. | | | | Children aged 5-9 years,  children aged 1-4 and 10+ opportunistically examined | Trachoma – follicular (TF -WHO grading) + PCR | | | | | 2019 | | | 8.6 |
|  |  |  |  |  |  |  |  |  |  |  |  |  |  |  | 2020 | | | 5.6 |
|  |  |  |  |  |  |  |  |  |  |  |  |  |  |  | 2021 | | | 8.9 |
| **Design** | | | | **Period** | **Location** | | **Population** | | | | | **Case definition** | | **Annual Incidence (%)** | | | | |
| **Incidence of visual impairment due to cataract, diabetic retinopathy and trachoma in indigenous Australians within central Australia: The Central Australian Ocular Health Study**  Landers J et al., 2012 (32) | | | | | | | | | | | | | | | | | | |
| Prospective population-based longitudinal cohort study  of clinic attendees,  point prevalence | | | | July 2005 – June 2008 | Central Australian Statistical Local Area, Northern Territory (very remote) | | 1, 884 Indigenous Australians aged ≥ 20 years residing in 30 remote communities who presented to scheduled eye-clinics | | | | | Trachoma (WHO grading) | | | 0.65 | | | |

Supplementary table 7: Characteristics of miscellaneous trachoma prevalence studies not included in national surveillance or large population-based surveys.

## Appendix E – trachoma control strategies

| () |
| --- |

Supplementary table 8: Community drug administration strategies and change in active trachoma prevalence. Adapted from Liu L, et al. PLoS Negl Trop Dis 2016;10:e0004921, and Lansingh VC, et al. Int Ophthalmol 2010;30:489–497.

| **Year** | **Population (n)** | Total treatment coverage (%) | NT | WA | SA |
| --- | --- | --- | --- | --- | --- |
| **2010** | 735 | 57 | 41 | 98 |  |
|  | 2, 851 | 72 | 67 | 89 |  |
| **2011** | 485 | 88 | 82 | 97 | 94 |
|  | 9, 509 | 65 | 82 | 85 | 94 |
| **2012** | 301 | 95 | 98 | 93 | 100 |
|  | 5, 046 | 81 | 79 | 93 | 97 |
| **2013** | 176 | 99 | 100 | 99 | 100 |
|  | 12, 596 | 80 | 79 | 93 | 98 |

Supplementary table 9: Azithromycin treatment coverage among children aged 1–14 years with trachoma, derived from Australian Trachoma Surveillance Annual Reports (2010–2013).

Key: NT, Northern Territory; WA, Western Australia; SA, South Australia. Note – grey shading indicates data not reported. Rows represent active cases (first row per year) and estimated contacts (second row per year).

## Appendix F - Non-trachomatous corneal vision loss

| **Design** | | **Period** | **Location** | **Population** | **Case definition** | **Prevalence estimate (%)** |
| --- | --- | --- | --- | --- | --- | --- |
| **Prevalence and causes of visual impairment in Indigenous Australians within Central Australia: The Central Australian Ocular Health Study**  Landers J et al., 2010 (30) | | | | | | |
| Prospective population-based longitudinal cohort study  of clinic attendees,  point prevalence | July 2005 – June 2008 | | Central Australian Statistical Local Area, Northern Territory (very remote) | 1, 884 Indigenous Australians aged ≥ 20 years residing in 30 remote communities who presented to scheduled eye-clinics | Bilateral visual impairment (VA < 6/12 OU) | 19.4 |
|  |  |  |  |  | Bilateral visual impairment due to corneal pathology (non-trachomatous) | 1.6 |
|  |  |  |  |  | Bilateral blindness (VA <6/60 OU) | 2.8 |
|  |  |  |  |  | Bilateral blindness due to corneal pathology (non-trachomatous) | 7.5 |
| **Prevalence and Causes of Unilateral Vision Impairment and Unilateral Blindness in Australia: The National Eye Health Survey**  Foreman J et al, 2018 (29) | | | | | | |
| Population-based cross-sectional survey; multistage random-cluster sampling across 30 sites stratified by remoteness (national) | Mar 2015 – Apr 2016 | | Australia-wide, 30 randomly selected clusters spanning all remoteness strata) | Indigenous Australians ≥40 years (and non-Indigenous ≥50 years;  n = 1,738 Indigenous Australians | Unilateral visual impairment (VA < 6/12 in worse eye) due to any corneal pathology | Indigenous  0.5 |
|  |  |  |  |  |  | Non-Indigenous  0.4 |
|  |  |  |  |  | Unilateral blindness (VA <6/60 in worse eye) due to any corneal pathology | Indigenous  16.7 |
|  |  |  |  |  |  | Non-Indigenous  2.1 |

Supplementary table 10: Vision loss prevalence not attributable to trachoma or trichiasis. Adapted from Landers J, et al. British Journal of Ophthalmology. 2010; 94:1140–1144, and Foreman J, et al. JAMA Ophthalmology. 2018; 136:240–248 (30, 29).
